# Depression among people with chronic skin disease at Boru Meda Hospital in Northeast Ethiopia

**DOI:** 10.1371/journal.pone.0282022

**Published:** 2023-02-24

**Authors:** Yasin Nurye, Minale Tareke, Meseret Tadesse, Maregu Shegaw, Tesfa Mekonen

**Affiliations:** 1 Department of Psychiatry, Boru Meda General Hospital, Dessie, Ethiopia; 2 Department of Psychiatry, College of Medicine and Health Science, Bahir Dar University, Bahir Dar, Ethiopia; 3 Department of Psychiatry, School of Nursing and Midwifery, College of Medicine and Health Science, Wollo University, Dessie, Ethiopia; 4 School of Psychology, The University of Queensland, Brisbane, Australia; 5 National Centre for Youth Substance Use Research, The University of Queensland, Brisbane, Australia; University of Otago, NEW ZEALAND

## Abstract

**Background:**

The comorbidity of depression with chronic skin disease negatively affects the quality of life and disease prognosis, creating an immense burden on patients, families, and the wider community. However, there are limited studies conducted on the prevalence of depression and associated factors among people with chronic skin disease in Ethiopia.

**Objective:**

This study aimed to assess the prevalence and associated factors of depression among people with chronic skin disease at Boru Meda Hospital, Northeast Ethiopia.

**Methods:**

An institutional-based cross-sectional study was carried out from March 10- April 18, 2021, among a total of 381 people with chronic skin disease. The Patient Health Questioner-9 was used to assess depression. A logistic regression analysis model with an adjusted odds ratio was used to assess the strength of associations between the outcome and predictor variables. P-value < 0.05 was considered statistically significant.

**Result:**

The magnitude of depression among people with chronic skin disease was 23.6% (95%Cl: 19.8%, 28.6%). We identified significantly increased odds of depression among participants with rural residence (AOR = 3.45, 95% CI: 1.64, 7.28), duration of illness above 5 years (AOR = 3.59, 95% CI: 1.31, 9.85), comorbid medical illness AOR = 2.51, 95% CI: 1.06, 5.98), family history of mental illness (AOR = 3.39, 95% CI: 1.11, 10.41), non-adherence to chronic skin disease medications (AOR = 3.53, 95% CI: 1.20, 10.41), low self-image (AOR = 4.69, 95% CI: 2.25, 9.77), and perceived stigma (AOR = 4.61, 95% CI: 2.14, 9.92).

**Conclusion:**

Depression was common among patients with chronic skin diseases. This study has indicated a need for proper screening of depression in the current medical treatment of patients with chronic skin disease in Boru Meda Hospital, Northeast Ethiopia.

## Introduction

The skin carries immense psychological significance in human life. As such, any disfiguring skin disorders have negative impact on mental health of individuals [1]. Chronic skin diseases including psoriasis, atopic dermatitis, vitiligo, leprosy, and cutaneous leishmaniasis [2, 3] have a devastating effect on a person’s physical, social, and psychological well-being. These psychosocial impacts consequently affect the quality of life [4–6]. The cosmetic disfiguration due to these chronic skin diseases can be a cause of stigma in social situations and patients can develop a low self-image that is directly associated with depression [5, 7–10].

On the other hand, depression is also one of the most common illnesses being the second leading cause of disability worldwide and a major contributor to suicide [11]. Depression is associated with chronic morbidity and mortality, which imposes a substantial burden in developed and developing countries [12] and is highly associated with chronic skin diseases [13].

Patients with chronic skin disease have a higher level of depression and lower level of self-esteem [5] most of the time, chronic skin disease patients who have comorbid depression have suicidal ideation [14]. The magnitude of depression among patients with chronic skin conditions ranges from 9–70% [10, 15, 16]. For instance, depression in psoriasis patients accounts for 42.33%, in vitiligo it is 46.3%, and in atopic dermatitis it is 35.45% [13].

For most patients with chronic skin diseases, their psychiatric diagnosis remains unrecognized and untreated [17]. This is because of the stigma of psychiatric illnesses, and patients prefer the treatment of their dermatological diseases rather than their psychiatric disorders which results in immense psychic and somatic suffering, social and occupational dysfunction, poor academic performance, drug abuse, suicide, and an increase in mortality [17]. Depression acts as a psychosocial stressor in initiating, exacerbating, and causing a relapse of skin problems. Conversely, it could be a consequence of dermatological disorders due to their long course and effects on self-esteem [18]. For example, depression significantly increased the risk of developing psoriasis and vitiligo [19]. On the other hand, vitiligo can lead to depression due to its cosmetically disfiguring visibility [20].

Depression also harms treatment adherence, quality of life, prognosis, and functional capacity, in people with chronic skin disease, resulting in poor vocational, and social functioning [21]. On the other hand, due to their chronic nature, effects on the individuals’ self-image, loss of hope of total recovery, and frequent recurrences, chronic skin diseases are considered one of the most important predisposing factors causing suicidal ideation [22].

The visibility of dermatological disease combined with its psychological impact often leads to feelings of embarrassment, decreased confidence, and fear of stigma [23]. Despite evidence of a high impact of depression among chronic skin disease patients, there is a lack of study on the prevalence and associated factors of depression among people with chronic skin disease in Ethiopia. Therefore, this study aimed to assess the prevalence and associated factors of depression among patients with chronic skin disease at Boru Meda Hospital in Northeast Ethiopia.

## Methods

### Study design and setting

An institutional-based cross-sectional study was conducted from March 10 -April 18, 2021, at Boru Meda General Hospital, Northeast- Ethiopia. The hospital currently has 40 beds for leprosy and other dermatology cases, in addition to other case teams. It also has three dermatology outpatient clinics with two dermatologists: a tropical dermatology professional and a health officer who has dermatology and leprosy training [24].

### Population

All patients with a chronic skin disease who were on follow-up were the source population of this study. People with chronic skin disease, who were 18 years and older, attended in dermatology clinic during the study period, and had taken medication for at least one month, were included.

### Sample size determination and sampling methods

We calculated the sample size using the single population proportion formula based on 34·6% proportion of mental distress among chronic skin diseases in Ethiopia [25], 95% confidence level, and 4% margin of error. Applying the correction formula and 10% non-response rate, the final sample size was 381.

A systematic random sampling method was used to employ participants who were attending Boru Meda Hospital. On average, 949 patients with chronic skin debases vested in the hospital in one month. Using the sampling fraction (K = 946/381), every other patient was selected to participate in the study. The first participant was selected by a lottery method.

### Data collection

The questionnaire included items to assess socio-demographic information, depression, perceived stigma, social support, self-image disturbance, medication adherence, clinical factors, psychosocial factors, and behavioral factors. Four health extension workers collected the data using face-to-face interviews. The data collection was supervised by a psychiatry nurse (bachelor’s degree holder). Training on the questionable and data collection proceed was provided for the supervisor and data collectors.

### Measurements

#### Chronic skin disease

An individual presented with at least one of the chronic skin diseases (Leprosy, Vitiligo, Atopic dermatitis, Psoriasis, cutaneous leishmaniasis) [5, 7–10] as reported in the patient medical record.

#### Depression

Participants were classified as having depression if they score 10 or more on the Patient Health Questionnaire-9 (PHQ-9). PHQ-9 is a self-administered screening measure that was developed as a brief screening tool for assessing depressive symptomatology in the primary care setting. The PHQ-9 items are rated on a four-point Likert scale (0 = not at all, 1 = several days, 2 = more than half of the days, and 3 = nearly every day) with the total sum score ranging from 0 to 27 (a high score indicates more severe depressive symptoms) [26]. PHQ-9 is validated in the Ethiopian context [27]. In this study, it has a good internal consistency (Cronbach’s alpha = 0.80).

#### Social support

The social support of patients was assessed using the Oslo social support scale (OSS-3). The OSS-3 provides a brief measure of social support [28]. In this study, it has a good internal consistency (Cronbach’s alpha = 0.75).

#### Medication adherence

The medication adherence of the participants for their skin disease treatment was assessed using ten binary Yes or No questions. A total score of 6 or more indicates good adherence, and less than 6 indicates non-adherence [29]. In this study, it has good internal consistency (Cronbach’s alpha = 0.86).

#### Quality of life

Quality of life was assessed using the dermatological life quality index (DLQI). The sum scores of DLQI rages from 0 to 30. Participants who scored ≥10 from the total score of 0 to 30 using the Dermatological Life Quality Index (DLQI) were considered as poor quality of life [30]. DLQI had an inter-item correlation average of 0.44 and Cronbach’s alpha of 0.90 in the current study.

#### Self-image

Self-image was assessed using the standardized 7-item body-image disturbance questionnaire (dermatologic version) (BIDQ). Patients with a chronic skin disease who score above the mean score (14.51) reflect greater body image disturbance [31].

#### Current use of a substance

The use of a specific substance like (alcohol, khat, cigarettes, and other illicit substances) for non-medical purposes in the last three months [32].

#### Ever use of a substance

The use of a specific substance like (alcohol, khat, cigarettes, and other illicit substances) for non-medical purposes at least once in lifetime [32].

#### Perceived stigma

Perceived stigma was assessed by a 6-item perceived stigmatization scale for skin disease. It has a 4-point Likert scale (0 = not at all, 1 = sometimes, 2 = very often, 3 = always) with a total score ranging from 0 to 18. At least one positive score among the 6-items were considered as having a high perceived stigma scale [33].

#### Family monthly income

Based on the World Bank poverty line cut point, those who have an average monthly family income of < 1.9 dollars per day (< 2394 Ethiopian Birr per month) are below the poverty line and those who have an income of ≥1.9 dollars per day (>2394 Ethiopian Birr per month) are above the poverty line [34].

### Data processing and analysis

The data were entered into EpiData version 4.6.02 and exported to SPSS version 25 for processing and analysis. All variables with a p-value of less than 0.2 in the bivariate logistic regression analysis were entered into the multivariable logistic regression model to identify factors associated with depression. The adjusted odds ratios (AORs) with 95% confidence intervals were used to assess the strength of associations between the outcome and predictor variables. The p-value of <0.05 was considered significant. The stepwise backward elimination (likelihood ratio) variable selection method was used in the multi-variable logistic regression model.

### Ethics approval and consent to participate

Ethical clearance was obtained from the ethical review board of Bahir Dar University, College of Medicine and Health Science. A permission letter was obtained from Amhara National Regional State public health institute and Boru Meda Hospital. Informed consent was obtained from each respondent after a detailed explanation of the study objective. The right to withdraw from the research process at any point in time was respected. Privacy and confidentiality were maintained throughout the study. For chronic skin disease patients, those who had suicidal ideas were advised and an immediate referral to a psychiatric clinic was provided. At the same time, social distance and infection prevention were maintained to prevent COVID-19 transmission.

## Results

A total of 373 people with chronic skin disease participated in this study, giving a 98% response rate. Three participants discontinued the interview, and five questionnaires were discarded because of incomplete data and there is no missing data in the table.

### Socio-demographic characteristics of respondents

The median age of the participants was 32 years (inter-quartile range of 23 years) and 188 (50.4%) of them were females. More than half of the participants 220 (59.0%) reside in rural areas. (Table 1).

**Table 1:** Description of socio-demographic characteristics of the respondents depression satus with chronic skin disease at Boru Meda Hospital, Northeast Ethiopia, 2021 (N = 373).

Variables	Category	Depression		P-value
		No = 285 N (%)	Yes = 88 N (%)	
**Sex**	Male	157(84.9)	28(15.1)	
	Female	128(68.1)	60(31.9)	0.001
**Age**	18–27 years	127(85.8)	21(14.2)	
	28–37 years	67 (85.9)	11(14.1)	
	38–47 years	38 (62.3)	23(37.7)	
	≥48years	53 (61.6)	33(38.4)	0.001
**Educational status**	Illiterate	68 (63.6)	39(36.4)	0.001
	Primary education	85 (74.6)	29(25.4)	
	secondary and preparatory	65 (91.5)	6 (8.5)	
	College and above	67 (82.7)	14(17.3)	
**Resident**	Rural	147(66.8)	73(33.2)	0.001
	Urban	138(90.2)	15(9.8)	
**Duration of illness**	Less than a year	76 (81.7)	17(18.3)	
	1 up to 5 years	177(84.3)	33(15.7)	
	Above 5 years	32 (45.7)	38(54.3)	0.001

### Clinical and psycho-social related factors

About 118 (31.6%) of participants had the diagnosis of cutaneous leishmaniasis, 92 (24.7%) had atopic dermatitis. In the psycho-social factors, 170 (45.6%) participants had a poor self-image, and almost half of the participants, 181 (48.5%) had faced feelings of perceived stigma (Table 2).

**Table 2:** Distributions of clinical and psycho-social characteristics of respondants depression status with chronic skin disease attending at Boru Meda General Hospitals, Northeast Ethiopia (N = 373).

Variables	Category	Depression		P- value
		No = 285 N (%)	Yes = 88 N (%)	
Comorbid medical illness	Yes	32 (52.5)	29(47.5)	0.001
	No	253(81.1)	59(18.9)	
Family history of mental illness	No	274(80.1)	68(19.9)	
	Yes	11 (35.5)	20(64.5)	0.001
medication adherence	Poor	12 (29.3)	29(70.7)	0.001
	Good	273(82.2)	59(17.8)	
Self-image	Good	185(91.1)	18(8.9)	
	Poor	100(58.8)	70(41.2)	0.001
Quality of life	Good	256(83.1)	52(16.9)	
	Poor	29 (44.6)	36(55.4)	0.001
Perceived stigma	Have no	165(91.2)	16(8.8)	
	Have	120(62.5)	72(37.5)	0.001
Social support	Poor	76 (60.3)	50(39.7)	0.001
	intermediate	160(86.0)	26(14.0)	
	Strong	49 (80.3)	12(19.7)	
Lifetime alcohol used	Yes	22 (53.7)	19(46.3)	0.001
	No	263(79.2)	69(20.8)	
Lifetime use of khat	Yes	101(70.1)	43(29.9)	0.024
	No	184(80.3)	45(19.7)	

### Substance use behaviors of participants

The lifetime use of substance from this study was 217 (58.2%). However, only 128 (34.3%) of participants used substances in the last 3 months. From the substance users in the last 3 months, 53 (14.2%) of them used khat and 78 (20.9%) used tobacco.

### Magnitude of depression

The magnitude of depression among people with chronic skin disease was found to be 23.6% (95%Cl: 19.8, 28.6). Among those who had depression, 80 (21.4%) had moderate depression and8(2.1%) had moderately severe depression. Of the total participants who reported depression, 60 (68.2%) were females.

### Factors associated with depression

In the multivariable analysis, residing in rural areas (AOR = 3.45, 95% CI: 1.64–7.28), duration of illness above 5 years (AOR = 3.59, 95% CI: 1.31–9.85), comorbid medical illness (AOR = 2.51, 95% CI: 1.06–5.98), family history of mental illness (AOR = 3.39, 95% CI: 1.11–10.41), non-adherence to chronic skin disease medications (AOR = 3.53, 95% CI: 1.20–10.41), low self-image (AOR = 4.69, 95% CI: 2.25–9.77), and perceived stigma (AOR = 4.61, 95% CI: 2.14–9.92) were found to be significantly associated with depression (Table 3).

**Table 3:** Bivariate and multivariable logistic regression: Factor associated with depression among people with chronic skin disease attending at Boru Meda Hospital, Northeast Ethiopia (N = 373).

Variables		Depression		COR (95%Cl)	AOR (95%Cl)
		No	Yes		
Resident	Rural	147	73	4.57(2.50–8.34)	3.45(1.64–7.28)
	Urban	138	15	1.00	1.00
Duration of illness	Less than a year	76	17	1.00	1.00
	1 up to 5 years	177	33	0.83(0.44–1.59)	0.60(0.24–1.1.5)
	Above 5 years	32	38	5.31(2.62–10.75)	3.59(1.31–9.85)
Comorbid medical illness	Yes	32	29	3.89(2.18–6.92)	2.51(1.06–5.98)
	No	253	59	1.00	1.00
Family history of mental illness	No	274	68	1.00	1.00
	Yes	11	20	7.33(3.35–16.02)	3.39 (1.11–10.41)
Medication adherence	Poor	12	29	11.18(5.39, 23.19)	3.53(1.20–10.41)
	Good	273	59	1.00	1.00
Self-image	Good	185	18	1.00	1.00
	Poor	100	70	7.19(4.06–12.75)	4.69(2.25–9.77)
Quality of life	Good	256	52	1.00	1.00
	Poor	29	36	6.11(3.45–10.84)	1.61(0.67–3.89)
Perceived stigma	No	165	16	1.00	1.00
	Yes	120	72	6.19(3.43–11.17)	4.61(2.14–9.92)
Social support	Poor	76	50	2.69(1.30–5.55)	2.78(0.98–7.89)
	Intermediate	160	26	0.66(.31–1.41)	1.52 (0.54–4.30)
	Strong	49	12	1.00	1.00
Lifetime alcohol used	Yes	22	19	3.52(1.81–6.81)	2.51(0.88–7.18)
	No	263	69	1.00	1.00

1 = reference group, COR = Crude Odds Ratio, AOR = Adjusted odds ratio, CI = 95% Confidence Interval

## Discussion

The prevalence of Depression among people with chronic skin diseases was 23.6% (19.6–28.2%). Depression was found to be higher among participants with rural residence, duration of illness above five years, poor medication adherence, comorbid medical illness, family history of mental illness, poor self-image, and high perceived stigma. This 23.6% magnitude of depression might be due to the vulnerability of individuals with chronic skin disease to various psychosocial stressors such as perceived stigma and lower self-image [35]. These psychosocial stressors are also reported in the current study, where 48.5% and 45.6% of participants reported perceived stigma and poor self-image, respectively. Individuals with chronic skin disease have also reported noncompliance with their medication, which has caused their illness to be prolonged and aggravated [36]. Depression can also develop as a result of the long-term effects of low self-esteem and physical appearance [37].

Our result is in line with studies in Muscat, Oman 24% [38], Sudan 21.9% [39], Canada 22.2% [40], America 20.1% [41], and North India 26.8% [42]. But, the current study result was higher than the studies done in Saudi Arabia 15.8% [37], Europe 10.1% [14], and Thailand 13.5% [43]. On the other hand, the prevalence of depression in the current study is lower than the studies done in Nigeria 49% [1], Egypt 39.25% [13], Iran 67% [44], and Germany 42.5% [45]. This might be because of the socio-cultural differences, and the lower number of study participants in these studies compared to the current study. The former studies were done mostly in high-income countries where most participants were educated, had good economic status, and living conditions. However, the current study was done in a developing country where the majority of participants were lower educated, low income, and most of them lived in rural areas.

Participants who reside in rural areas were 3.45 times more likely to have depression [AOR = 3.45, 95% CI: 1.64, 7.28] compared to those who live in urban areas. This is supported by the study done in Ethiopia [35]. Rural residents commonly have less access to primary health care, specialists, health-related technologies, and other health and social services than persons in urban areas [46, 47]. As a result, people with chronic skin disease in rural areas may not be treated on time, leading to complications such as depression. This was also supported by the study done in the United States [48].

Those with a duration of illness above five years were 3.59 times more likely to have depression [AOR = 3.59, 95% CI: 1.31, 9.85] than those with less than one-year duration. This is consistent with a study done in Nigeria [36]. This may be due to the physical and emotional toll of living with visible skin condition for a prolonged time. For instance, comorbidieteis of medical conditions are common in chronic skin diseases leading to a higher risk of depression [49]. This is also reported in our study that participants who had comorbid medical illnesses were 2.51 times more likely to have depression [AOR = 2.51, 95% CI: 1.06, 5.98]. Family history of mental illness was also associated with higher odds of depression [AOR = 3.39, 95% CI: 1.11, 10.41] These findings are consistent with a study done in Oman [38].

In this study, participants with a lower self-image were 4.69 times more likely to have depression [AOR = 4.69, 95% CI: 2.25, 9.77]than those with a good self-image. This is consistent with the study conducted in Poland [50, 51]. This could be due to individuals having long-term visible changes on their appearance may have low self-acceptance, low self-esteem, and poor quality of life [51–53]. Additionally, the cosmetic disfigurement of the exposed areas, causes individuals to limit social gatherings and develop a low body image, leading to social withdrawal, severe depression, and suicidal attempts [54].

In the current study, patients who reported high perceived stigma had 4.61 times [AOR = 4.61, 95% CI: 2.14, 9.92] odds of depression compared to those who had not reported perceived stigma. This is in line with the study done at Alert hospital in Addis Ababa, Ethiopia [35]. This might be due to individuals with perceived stigma restricting themselves from work opportunities, and social activities and being perceived as having less respect by the community. Such people experience low self-esteem and a poor quality of life, which leads to significantly higher depression [54, 55]. In addition, having poor medication adherence was 3.53 times [AOR = 3.53, 95% CI: 1.20, 10.41] more likely to associate with depression. This increment could be because of the recurrence and poor prognosis of skin diseases due to medication non-adherence [56]. This finding is supported by a study in Nigeria [36].

### Limitations

This study has some limitations. Even though PHQ-9 is a locally validated tool used to assess depressive symptoms, it is not a diagnostic tool. This study also lacks more specificity to include some factors like disability and income, which were not assessed with a standardized tool.

Social desirability bias may be a concern for this study since the skin is a sensitive issue and participants were interviewed face-to-face. participants may answer questions in a manner they perceived as most desirable to the interviewer.

## Conclusion

This study showed that the overall prevalence of depression among chronic skin disease patients in Boru Meda Hospital was common. It was also found that residing in rural areas, duration of illness more than five years, comorbid medical illness, family history of mental illness, non-adherence to medication, poor self-image, and perceived stigma were factors that were significantly associated with depression. As a result, a psychosocial assessment should be addressed in the evaluation and treatment of chronic skin diseases. The findings of this study will provide information for future studies on the association between depression and skin disease.

## Supporting information

S1 Data (SAV)

## Abbreviations

AD: Atopic Dermatitis
AOR: Adjusted odds ratio
BDI: Beck Depression Inventory
BIDQ: Body-Image Disturbance Questionnaire
CI: Confidence Interval
DLQI: Dermatological Life Quality Index
